# Immunomodulatory Effects of Diet and Nutrients in Systemic Lupus Erythematosus (SLE): A Systematic Review

**DOI:** 10.3389/fimmu.2020.01477

**Published:** 2020-07-22

**Authors:** Md Asiful Islam, Shahad Saif Khandker, Przemysław J. Kotyla, Rosline Hassan

**Affiliations:** ^1^Department of Haematology, School of Medical Sciences, Universiti Sains Malaysia, Kubang Kerian, Malaysia; ^2^Department of Biochemistry and Molecular Biology, Jahangirnagar University, Dhaka, Bangladesh; ^3^Department of Internal Medicine, Rheumatology and Clinical Immunology, Medical Faculty in Katowice, Medical University of Silesia, Katowice, Poland

**Keywords:** diet, nutrients, autoimmune diseases, systemic lupus erythematosus (SLE), polyunsaturated fatty acids, vitamins, minerals, polyphenols

## Abstract

Systemic lupus erythematosus (SLE) is an autoimmune disease characterized by multiple organ involvement, including the skin, joints, kidneys, lungs, central nervous system and the haematopoietic system, with a large number of complications. Despite years of study, the etiology of SLE remains unclear; thus, safe and specifically targeted therapies are lacking. In the last 20 years, researchers have explored the potential of nutritional factors on SLE and have suggested complementary treatment options through diet. This study systematically reviews and evaluates the clinical and preclinical scientific evidence of diet and dietary supplementation that either alleviate or exacerbate the symptoms of SLE. For this review, a systematic literature search was conducted using PubMed, Scopus and Google Scholar databases only for articles written in the English language. Based on the currently published literature, it was observed that a low-calorie and low-protein diet with high contents of fiber, polyunsaturated fatty acids, vitamins, minerals and polyphenols contain sufficient potential macronutrients and micronutrients to regulate the activity of the overall disease by modulating the inflammation and immune functions of SLE.

## Introduction

Systemic lupus erythematosus (SLE), an autoimmune disease, is characterized by abnormal inflammatory responses due to complex, aberrant humoral and cellular immune responses. The pathogenesis of SLE is largely unknown; however, data from the literature suggest that manifestation of this disease is the result of several environmental, hormonal, and nutritional factors that, in predisposed subjects, contribute to impaired cellular, and humoral immune responses (1–3). Accordingly, the presence of autoantibodies is nearly universal among patients with SLE. Indeed, having antinuclear antibodies specifically against double-stranded DNA (dsDNA) is a hallmark classification criterion for SLE; this and other clinical and immunological criteria must be satisfied for SLE classification as proposed by the European League Against Rheumatism and American College of Rheumatology (4). Autoantibodies also contribute to the synthesis of multiple immune complexes and exert direct cytotoxic effects. As a result, SLE affects the whole body with no system spared; finally, damage occurs in multiple organs including the kidneys, central nervous system (CNS), skin, joints and haematopoietic system (5, 6). Although SLE represents a prototypical autoimmune disorder, its prevalence is relatively low, estimated between 6.5 and 178.0 per 100,000 people, with an incidence ranging from 0.3 to 23.7 per 100,000 people per year (7).

The contribution of lifestyle-associated factors is still a matter of controversy in SLE; however, dietary habits and dietary-related microbiome composition are receiving more attention from researchers (8, 9). Indeed, some SLE-related clinical features are associated with nutrition; perhaps not as an aetiological factor but as a clinical repercussion (10). Thus, SLE represents a mosaic of metabolic changes and mineral and vitamin deficiencies superimposed by the systemic presentation of arthritis, nephritis, vascular events and organ damage to the heart, CNS, kidneys, and skin, which contribute to increases in the morbidity and mortality of these patients (6, 11, 12). In the last two decades, many clinical, and preclinical studies have investigated the impact of diet and nutrients on SLE inflammatory response and disease activity. This has become an important highlighted topic and remains under investigation by many researchers. Nutritional therapy including restrictions on carbohydrate and protein and the use of nutritional supplements (*i.e*., vitamins, minerals and polyphenols) is a promising way to control inflammatory responses in SLE (8, 13). Nutritional supplements may exert potentially prophylactic effects with fewer or no side effects than those of the classic pharmacological therapies besides reducing co-morbidities and improving the quality of life of patients with SLE.

As a broad range of evidence has demonstrated that some diets and nutrients have antioxidant, anti-inflammatory and immunomodulatory effects on immunoinflammatory diseases, the present study evaluates the impact of diet and nutrients on SLE based on the existing *in vivo* studies in animal models and human subjects.

## Methods: Search Strategy

**Figure 1:**
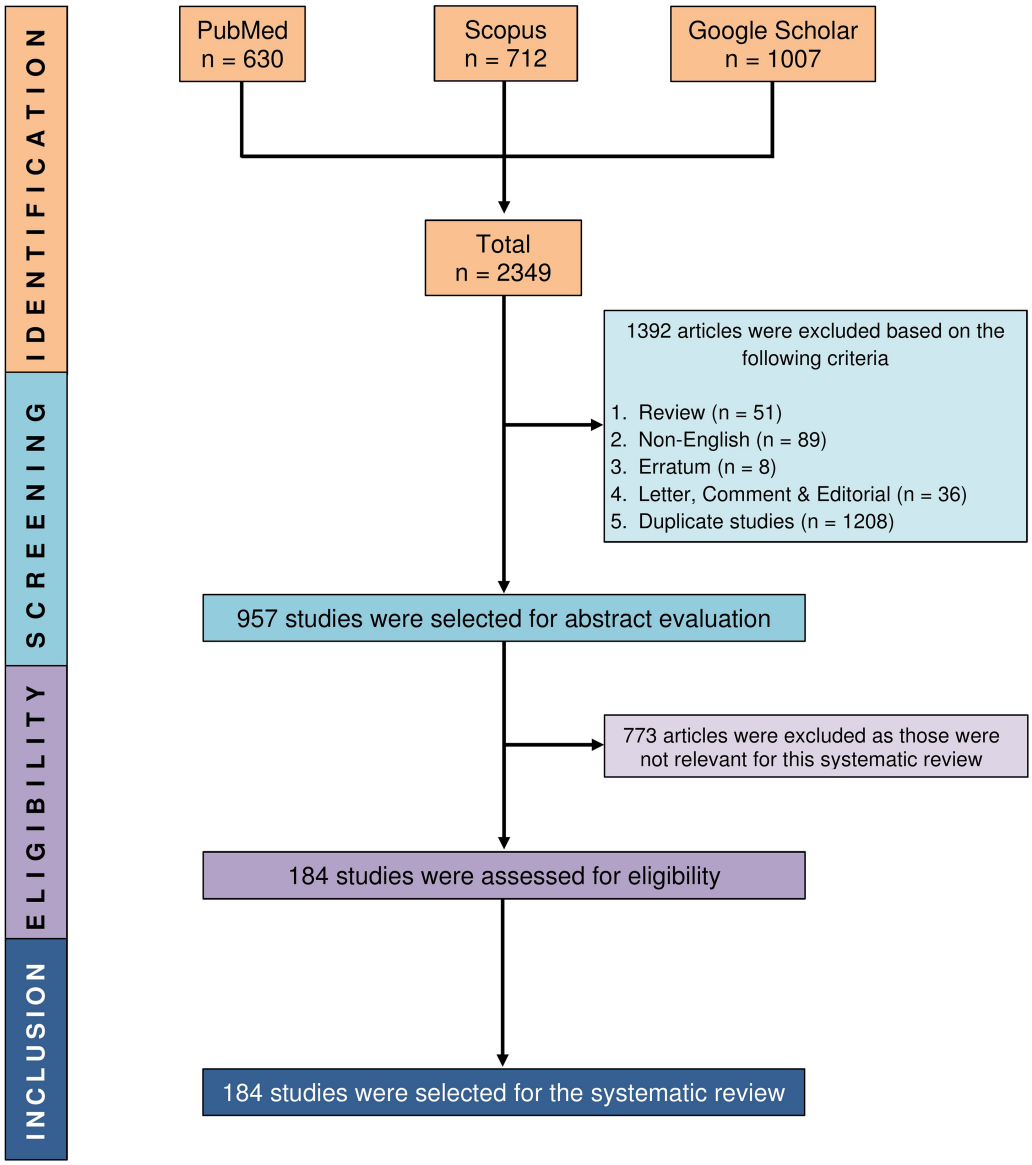
PRISMA flow diagram showing the process of selecting relevant studies.

A systematic search strategy was developed by combining the terms SLE, “Systemic lupus erythematosus,” lupus, food, nutrient^*^, diet, intake, antioxidant^*^, nutrition^*^, benefit^*^, nutrition^*^, physicochemical, dietary, bioactive, composition, supplement^*^, vitamin^*^, mineral^*^, phenol^*^, “olive oil,” and curcumin, where quotations represented an exact term and an asterisk (*) denoted a root word or wildcard term. PubMed, Scopus, and Google Scholar electronic databases were searched combining the appropriate keywords with Boolean logical operators “AND” and “OR” using “Advanced” and “Expert” search options (Appendix). Only English-language articles were searched. There was no year restriction, and the final systematic search was conducted on 22 December 2019. Review articles, non-English articles, errata, letters, comments, editorials, and duplicate articles among different databases were excluded. Duplicate studies that resulted from different electronic databases were removed and managed by EndNote software (version X8). Study selection methodology is illustrated in Figure 1.

## Macronutrients

Macronutrients represent the group of environmental substances widely used by organisms for vital processes such as growth, body development, and bodily functions. Several theories have described the effects of macronutrient- or macronutrient-derived molecules including glucose, amino acids, and fatty acids on body weight regulation, maintenance of homeostasis, and the immune response.

### Carbohydrates

Carbohydrates are among the macronutrients that provide energy and, when consumed in excess, contribute to increased energy storage and subsequent weight gain. Although there is no clear evidence that altering the proportion of total carbohydrate in the diet is an important determinant of energy intake, nutritional imbalance, and excess carbohydrate dietary intake have been suggested as risk factors that exacerbate clinical manifestations of several autoimmune diseases such as rheumatoid arthritis and SLE (14). Obesity is a well-known risk factor for low-grade inflammation characterized by activation of several pathways involved in the expression of inflammatory cytokines such as tumor necrosis factor-alpha (TNF-α) and interleukin (IL)-6. Activation of these proinflammatory pathways significantly contributes to the perpetuation of the inflammatory response, which are at least partly responsible for the severe co-morbidities seen in SLE patients (15). Indeed, patients with SLE are characterized by a high-risk of developing metabolic syndrome, insulin resistance and type 2 diabetes mellitus (T2DM) (16), which can contribute to increased risk of developing cardiovascular co-morbidities, a leading cause of premature death in SLE patients (17).

Indeed, several studies have shown that up to 35% of SLE patients are overweight and 39% are obese, and these patients are characterized by a higher concentration of inflammatory markers including C-reactive protein (CRP) (18, 19). Recent studies have suggested that obesity is associated independently with SLE disease activity (20, 21). Corticosteroids remain the first choice of treatment for SLE, but their administration is linked to excess weight gain and the development of corticosteroid-induced diabetes. Obesity was detected as an independent risk factor in worsening the functional capacity, fatigue, and inflammation status of patients with SLE (20, 22–24).

#### Mouse Models

Mouse models provide excellent insight into the pathogenesis of SLE and the observation of dietary-induced changes. Notably, the restriction of calorie intake leads to substantial changes in the immune response. For example, in a study with a lupus-prone mouse model (NZB/NZW F1), calorie intake restriction effectively delayed the onset of proteinuria and significantly decreased serum levels of anti-dsDNA antibodies. Calorie restriction also had a significant impact on the B-cell population, resulting in a reduction of their frequency and activity. Parallel to this, a decline in CD8^+^ T cells and a higher proportion of naïve CD4^+^ and CD8^+^ T cells have been observed (25). This finding is of special importance as it shows the direct impact of calorie restriction on B-cell and T-cell compartments, key immunocompetent cell compartments involved directly in SLE pathogenesis (26, 27). In another SLE-prone mouse model, when a 40% calorie-restricted diet was provided, B:T cell and CD4^+^:CD8^+^ T-cell ratios were significantly lowered compared with the control group (28). This suggests a decline in the predominance of abnormally activated T cells and regulatory T (Treg) cells that potentially reduces disease activity.

The impact of calorie intake on autoimmune system functioning is not restricted to immune executive cells. At the end of the last century, Troyer et al. (29) showed that a calorie-restricted diet was successful in modulating the key inflammatory ligand platelet-derived growth factor (PDGF) subunit A and the thrombin receptor, resulting in the suppression of murine lupus nephritis. Quite recently, the direct mechanism of this finding has been explained, elucidating the role of PDGF as a growth factor that regulates cell proliferation and is responsible for mesangial proliferation, periostin formation and progressive glomerulosclerosis (30).

Less is known about the humoral response in this regard. The influence of calorie restriction on the expression of main cytokines and synthesis of immunoglobulin (Ig)G has been tested in NZB/NZW F1 mice. In the study, reduced calorie intake contributed to the reduction of *interferon (IFN)-*γ, *IL-10*, and *IL-12* mRNA expression besides the reduction of IgG secretion in the submandibular glands (31).

#### Human Study

During the last several decades, the prevalence of excess body weight has increased rapidly worldwide and is now recognized as a main public health crisis (32). Obesity has a strong impact on organism functioning and is linked to the development of all diseases of civilization, including metabolic syndrome, atherosclerosis and T2DM. Strong evidence also links obesity to many autoimmune disorders including SLE (8, 33, 34). Obesity in SLE patients is associated with a poorer outcome, higher disease activity and higher cumulative organ damage (34, 35). Therefore, the importance of prevention and treatment of obesity is widely acknowledged. Unfortunately, these recommendations are chiefly driven from cross-population studies; thus, data on the direct impact of body weight reduction on disease activity in SLE are limited.

The influence of body weight reduction on SLE activity has been addressed by Davies et al. (36). They enrolled 23 overweight female subjects (BMI: >25 kg/m^2^) with SLE who were on corticosteroid therapy. A 6-week controlled trial where a low glycaemic index diet (*n* = 11) or a calorie-restricted diet (*n* = 12) was implemented resulted in significant weight loss, reduction in waist and hip measurements and fatigue in both treatment groups. Of note, caloric restriction did not cause any flares of disease. However, these results must be interpreted with caution because they from only one study that addresses this issue, and the population studied was small. Nevertheless, it is surprising that such an important problem has not yet attracted greater attention, and more studies in this field are required. Presently, evidence supports the idea that a hypocaloric diet reduces the disease activity of SLE. By contrast, however, not all obese patients share inflammatory profiles, and not all individuals in a healthy weight range are metabolically healthy (37, 38). This underscores the need for proper nutrition or weight loss as only two factors of many for lupus patients (18). Lifestyle modifications such as meditation and exercise can also ameliorate lupus symptoms (39–43). Therefore, patients with SLE should maintain a balanced diet with caution to avoid excess daily calorie intake besides avoiding a sedentary lifestyle, especially in the case of obese patients or patients with a tendency toward obesity.

### Proteins

The restriction of dietary protein has been addressed in several studies in patients with SLE and animal models. These data should be interpreted in a wider context as diet composition rather than protein restriction alone may show beneficial effects on SLE course. As an example, a traditional Mediterranean diet provides protection from certain chronic diseases including autoimmune disorders. This diet consists of vegetables, fruits, nuts, grains, olive oils and fish with limited meat consumption. Reduction of protein intake may be a reasonable approach in cases of lupus nephropathy as high protein intake contributes to reduced renal filtration, directly leading to the progression of kidney damage (44). Indeed, Milovanov et al. (45) observed that early restriction of dietary protein (0.6 g/kg/day) slowed the decline in glomerular filtration rate in patients with SLE-induced chronic kidney disease. In a cross-sectional study on Brazilian juvenile subjects with SLE (*n* = 22), consistent bone mineral loss was detected when subjects were treated with excessive proteins (46).

It has also been postulated that not only proteins but also selected amino acids may influence the course of SLE. In a case-controlled study, levels of serum L-canavanine (a non-proteinogenic amino acid) was significantly high (*p* < 0.01) in a group of Mexican patients with SLE (*n* = 100) compared with those of healthy controls (*n* = 100). This was therefore postulated to be a risk factor in developing SLE (47).

Among the many mechanisms by which amino acids modulate the immune response, regulation of mechanistic target of rapamycin (mTOR) attracts special attention. The signaling of mTOR is recognized as the most important intracellular pathway that coordinates local nutrients and systemic energy status at the organismal and cellular levels (48). Moreover, it is deeply involved in the immune response; thus, any dysfunction in this pathway may result in an aberrant immune response and predisposition to the development of autoimmunity (49). The role of the mTOR pathway is usually discussed in the context of T-cell function (50). It has been shown recently that T-cell dysfunction observed in SLE is at least partially due to the activation of mTOR caused by reduced glutathione (GSH) depletion via mitochondrial hyperpolarisation (51, 52). It was established that supplementation with N-acetylcysteine (NAC), a precursor of GSH, significantly improved the activity of SLE disease by profoundly blocking mTOR activity (*p* < 0.0009) in T cells and reversing GSH depletion (53). Regarding the other amino acids, in case-controlled studies (54, 55), lower levels of tryptophan and higher levels of kynurenine (a metabolite of tryptophan) with activation of the kynurenine pathway were detected in patients with SLE. Indeed, an interventional study showed that NAC treatment significantly reduced the levels of kynurenine (*p* = 2.8 × 10^−7^) in patients with SLE (56). Therefore, supplementation with NAC or a GSH-rich diet and nutrients may exert a therapeutic role in the management of SLE.

Contrary to this, a diet low in phenylalanine and tyrosine showed a protective role against nephropathy (57) and reduced autoantibody production in a mouse model (NZB/W) of SLE (58). In another study, high L-arginine intake was associated with renal fibrosis and shortened the life span of MRL/*lpr* SLE mice (59).

Considering the role of proteins and amino acids, it should be emphasized that a diet of moderate protein intake is recommended, and a high-protein diet should be avoided especially by patients with lupus-related kidney diseases (*e.g.*, overt lupus nephropathy). Major sources of dietary proteins are shown in Table 1 based on the *United States Department of Agriculture (USDA) National Nutrient Database for Standard Reference* (60).

**Table 1:** Major nutrient sources related to systemic lupus erythematosus.

**Nutrients**	Sources^*^	
**MACRONUTRIENTS**		
Amino acids		Eggs, meat, dairy products, pulses/legumes, whole cereals, royal jelly, and seafoods.
Polyunsaturated fatty acids	Omega-3	Fish oil, krill oil, flaxseed oil, canola oil, soybean oil, olive oil, nuts, margarine, and fishes (*i.e*., salmon, tuna, sardine, herring, mackerel, sablefish, whitefish).
	Omega-6	Safflower oil, sunflower oil, soybean oil, maize oil, sesame oil, canola oil, corn oil, poppyseed oil, nuts, walnut oil, primrose oil, margarine, ruminant, meat, eggs, and milk.
Fiber		Beans, cereals, pulses/legumes, whole grains, vegetables, fruits, curry powder, cinnamon, dried rosemary, dried oregano, coriander seed, dried basil, chili powder, and cloves.
**MICRONUTRIENTS**		
Vitamins	A	Carrots, sweet potatoes, pumpkins, spinach, shallots, kale, pepper, liver, fish oil, various meats, and tropical fruits.
	B complex	Fortified cereals, peanut butter, potatoes, dried peppers, nuts, banana, avocado, eggs, chicken, various red meats, liver, mollusks, salmon, and sardine.
	C	Tangerine, orange juice, apple, papaya, guava, litchis, kiwi, broccoli, tomato, carrot, pepper, and whole cereals, green tea, coriander leaf.
	D	Sunlight exposure, eggs, liver, fatty fishes (*i.e*., salmon eel, mackerel, trout sturgeon, swordfish, and sardine), fish oil, cod liver, mushrooms, and supplemented dairy products.
	E	Wheat germ, sunflower oil and seeds, canola oil, soybean, whole cereals, nuts, almonds, peanut butter, milk, fish, spinach, pepper, and margarine.
Minerals	Calcium	Dairy products, dried basils, dried tofu, kale, soybean, spinach, sardine, and fortified whole cereals.
	Zinc	Mollusks, whole cereals, peanut butter, seeds, white beans, soybean, spinach, milk, beef, turkey, and lamb.
	Sodium	Table salt, soy sauce, salted fishes (*i.e*., mackerel and salmon), wasabi, salted tofu, chili powder, canned foods, and cheeses with salt.
	Selenium	Pike, carp, rainbow trout, mollusks, wheat germ, whole cereals, sunflower seeds, nuts, fish (tuna, cod, haddock, salmon, crayfish, herring), egg, chicken liver, turkey, lamb, beef, mustard seed, fortified flours and products, and ricotta.
	Iron	Fortified whole cereals, dried basil, dried spearmint, seaweed, cumin seed, fenugreek seed, turmeric, bay leaf, soybeans, kale, pulses/legumes, mollusks, various meats, spinach, and broccoli.
	Copper	Beans, sesame seeds, sunflower seeds, dried basil, lentils, mushrooms, seaweed, nuts, mollusks, various meats, and liver.
Polyphenols		Various fruits and vegetables (*i.e*., grapes, oranges, watermelon, kiwi, apple, tomato, lettuce, broccoli, asparagus, spinach, lentils, celery, parsley, thyme, and peppermint), green tea, coffee, walnut, green, and white beans, olive oil, chamomile olives, and legumes.

* *Based on the USDA, National Nutrient Database for Standard Reference 2018*.

### Essential Fatty Acids

**Figure 2:**
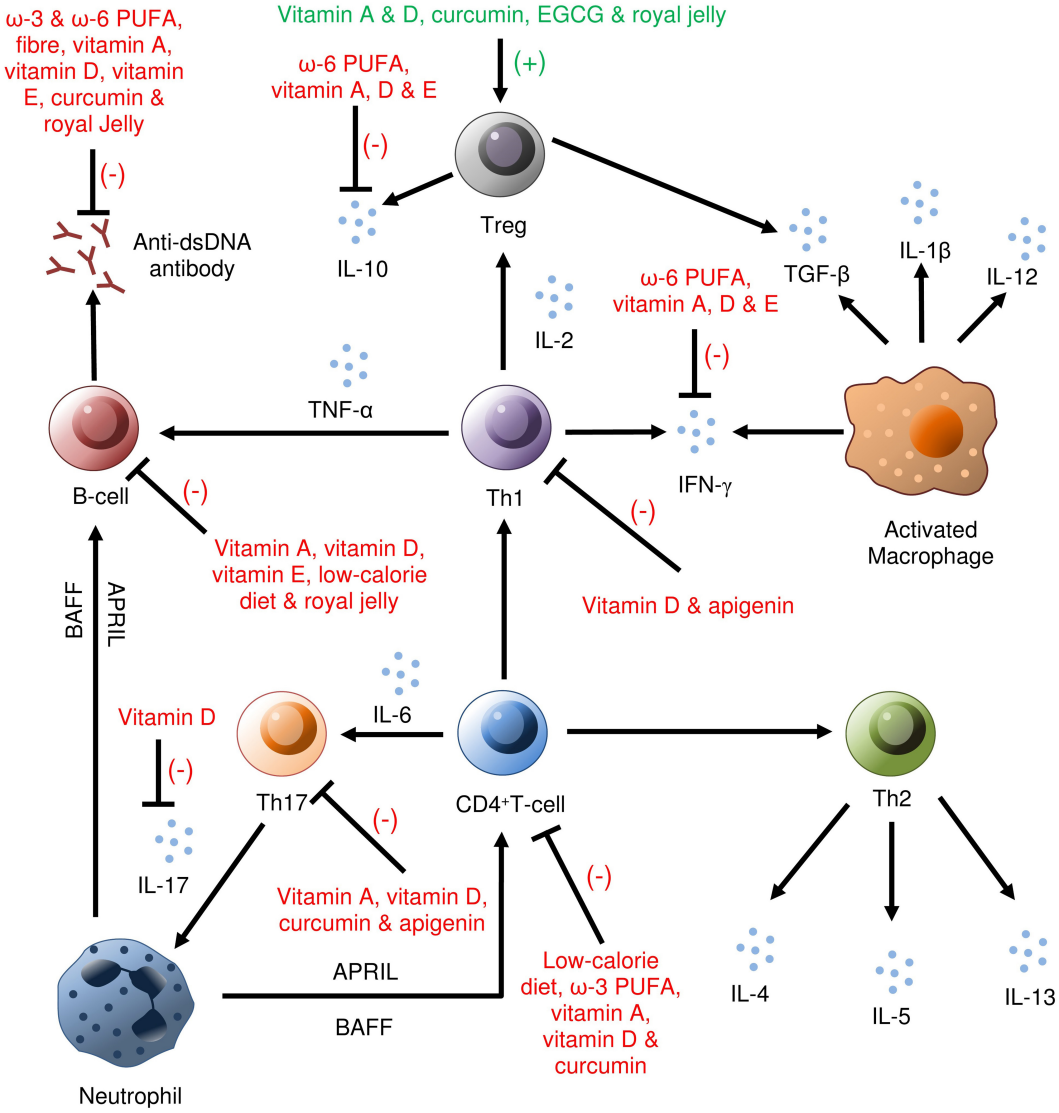
Immunomodulatory benefits of nutrients in SLE (based on the studies on human and animal models). In the immune-mediated pathogenesis of SLE, vitamin A inhibits anti-dsDNA antibodies, activation of B-cells, IFN-γ, CD4^+^ T-cell, Th17 cells, IL-10; vitamin D inhibits IL-10, IL-17, IFN-γ, B-cells, Th1, Th17, CD4^+^ T cells; ω-3 PUFAs inhibit anti-dsDNA antibodies, CD4^+^ T-cells and ω-6 PUFAs inhibit anti-dsDNA antibodies, IL-10, and IFN-γ. Vitamin A, vitamin D, curcumin, EGCG, and royal jelly were found to activate Treg cells. Anti-dsDNA, Anti-double-stranded DNA; PUFA, Polyunsaturated fatty acids; EGCG, Epigallocatechin-3-gallate.

Fatty acids (FAs), especially polyunsaturated FAs (PUFAs), are an effective and essential dietary factor for patients with SLE (61). Among PUFAs, omega-3 (ω-3) fatty acids [*i.e.*, docosahexaenoic acid (DHA) and eicosapentaenoic acid (EPA)] can reduce the level of inflammatory mediators (Figure 2) as well as CRP. EPA and DHA can further reduce lymphocyte proliferation, macrophage-mediated and cytotoxic T-cell-mediated cytotoxicity, synthesis of proinflammatory cytokines, and chemotaxis from monocytes and neutrophils.

In a lupus-prone mouse model, when animals were fed with DHA, IFN-related genes were suppressed besides reduced autoantibody levels and glomerulonephritis (62). In another lupus-prone mouse model (female NZBWF1), the autoimmune response (*i.e*., increased levels of proinflammatory cytokines) was triggered by airway silica exposure, resulting in glomerulonephritis, lung damage and autoantibody formation. It was established that an isocaloric diet containing DHA reduced cytokine and chemokine synthesis, lymphocyte infiltration and autoantibody synthesis by plasma cells in this model (63).

In the mid-1980s, Kelley et al. in his pioneer study (64) demonstrated the direct impact of a fish oil diet (rich in EPA) on prostanoid metabolism and function that ultimately led to reduction of the inflammatory response in MRL/*lpr* mice. Many years later, the mechanism of this phenomenon was explained. The direct anti-inflammatory effect of EPA was due to the inhibition of cyclooxygenase (COX)-1, a key enzyme in the prostanoid synthesis pathway (65). Parallel to the inhibition of COX, fish oil ω-3 PUFA suppressed autoantibody production and reduced the gene expression of inflammatory response-related products, especially in the spleen and kidney of a murine SLE model. These mechanisms ultimately lead to the inhibition of glomerulonephritis and inflammation and prolong the lifespan of lupus-prone mice (66, 67). Fish oil maintained the enzymatic ratios of reduced GSH to oxidized GSH (GSH:GSSG) and the antioxidant profile in lupus-prone aged B/W mice (68). Furthermore, DHA-enriched fish oil reduced IgG deposition and caspase 1 in the kidney and anti-dsDNA antibody production and lipopolysaccharide-induced IL-18 production in the serum. It downregulated genes related to CD4^+^ T cells and decreased the expression of *TNF-*α, *CD80, CXCR3, CTLA-4*, and various ILs (*i.e., IL-6, IL-10*, and *IL-18*) in the kidneys and spleens of SLE-prone mice (66, 67).

Translationally, these immunomodulatory mechanisms assist to minimize inflammatory responses in patients with SLE (69–71). Omega-3 PUFA from fish oil improved the endothelial function of patients with SLE in one study (72) but not in another (73). Besides their anti-inflammatory activity, dietary fish oil supplements also have played important roles in decreasing symptoms related to neuromotor and cardiovascular involvement and have improved conditions of fatigue and depression in patients with SLE (72, 74). Recently, in a double-blind randomized controlled trial, Seluang fish (*Rasbora* Spp.) oil (500 μL/day) reduced the inflammatory response in patients with SLE by increasing serum levels of vitamin D and reducing IL-1, IL-6, and IL-17 (75). Altogether, concentrated fish oil increased the activity of renal antioxidant enzymes (*i.e*., GSH peroxidase and catalase) and serum vitamin D levels and reduced IL-1, IL-6, IL-1β, IL-17, and TNF-α besides anti-dsDNA antibody production in the kidney, spleen and liver.

A similar conclusion comes from studies on linoleic acid (ω-6 PUFA). Conjugated linoleic acid (CLA, an isomer of linoleic acid) demonstrated significant efficacy in SLE-induced rats by hindering cytokine and autoantibody production by controlling splenomegaly. It also obstructed oxidative stress as well as the nuclear factor kappa B (NF-κB) pathway and thus improved SLE activity and minimized mortality rates (76–78). Moreover, CLA demonstrated beneficial effects in SLE by improving the lipid profile in animal models by exhibiting antioxidant and anti-sclerotic activities (76–78).

Extra virgin olive oil (EVOO), as a main source of unsaturated fatty acids, reduced disease activity in a pristane-induced SLE mouse model by influencing proinflammatory cytokine expression in the spleen and kidney besides regulating prostaglandin E_2_ levels in the kidney and matrix metalloproteinase-3 in the serum. The potential mechanisms by which EVOO may exert beneficial effects on SLE activity may involve such pathways as mitogen-activated protein kinase, Janus kinase/signal transducer and activator of transcription, NF-κB, NF-E2-related factor-2, and heme oxygenase-1 pathways (79).

Only two studies addressed the role of fatty acids in patients with SLE. In a cross-sectional study on women with SLE (*n* = 105), a diet with inflammatory potential (measured here as high dietary inflammatory index) was associated with a less-favorable lipid profile in patients with SLE (80). A population-based study (*n* = 456) suggested that a higher dietary intake of ω-3 fatty acids and lower ω-6:ω-3 ratios were favorably associated with self-reported lupus activity and sleep quality (81). This is an interesting finding that suggests a direct influence of a ω-3-rich diet on the disease activity and quality of life in patients with SLE. Among many mechanisms that may potentially explain this finding, the impact of EPA and DPA on the synthesis of serotonin has been recently proposed (82). Higher serotonin levels may simply alleviate dysfunction in the serotoninergic system, thereby contributing to the reduction of neuropsychiatric symptoms, depression and sleep disturbance (82).

Despite certain fatty acids significantly improving SLE conditions, some research has indicated ambiguity concerning their effectiveness in SLE (especially of ω-3 and ω-6 PUFAs), which are related to overconsumption, dose-dependent complexity, adverse immune responses and accelerated autoimmune symptoms; these should be carefully considered (83–85). Major sources of dietary PUFAs (ω-3 and ω-6), based on the *United States Department of Agriculture (USDA), National Nutrient Database for Standard Reference* (60), are shown in Table 1.

### Fiber

Dietary fiber consists of edible carbohydrate polymers with three or more monomeric units that are resistant to endogenous digestive enzymes and are thus neither hydrolysed nor absorbed in the small intestine (86). Although fiber is present in a wide range of plant-based food sources, consumption is low in Western countries, contributing to changes in gut microbiota that may influence the development of serious gastrointestinal, cardiovascular and autoimmune disorders (87). Indeed, Statovci et al. (88) observed that a westernized diet with low fiber stimulates pathogenic bacterial growth inside the gut. Other studies also found a connection between fiber intake and diversity of intestinal microbiota (89, 90). Low levels of short-chain fatty acids (SCFAs), an end product of fiber fermentation, due to lower availability of dietary fiber can result in inflammation and imbalance in innate and adaptive immunity (91, 92). Parallel to this dietary residue, complex carbohydrates (fiber) are substrates for fermentation that produces SCFAs, and these serve as an energy source for gut microbiota. Thus, any changes in fiber intake may lead to a reduction of the population of gut microbiota (dysbiosis). Dysbiosis is not an uncommon phenomenon in patients with SLE and can be partially explained by the insufficient intake of dietary fiber (93–97). In a prospective study on Japanese female patients with SLE (*n* = 43) (98), an inverse correlation was observed between high intake of dietary fiber and risk of active, possibly through improving overall immune functions and suppressing active inflammation.

Therefore, an adequate intake of dietary fiber is recommended in patients with SLE because of the beneficial effects of fiber in reducing the disease activity by decreasing serum levels of autoantibodies and inflammatory cytokines. The major sources of dietary fiber are shown in Table 1 based on the *United States Department of Agriculture (USDA), National Nutrient Database for Standard Reference* (60).

## Micronutrients

Micronutrients comprise chemical compounds and essential elements that are required by organisms in varying quantities (usually very low) throughout life to orchestrate a range of metabolic activities to maintain health. This term usually refers to the vitamins and minerals that cannot be synthesized by the body and must be derived from the diet (99). Micronutrients typically can be divided into four categories: water-soluble vitamins, fat-soluble vitamins, microminerals and trace minerals.

### Vitamin A

Vitamin A, a member of the fat-soluble vitamins, is an essential factor with multiple functions, including maintaining immune system, integrity and proper function besides acting upon nuclear retinoic acid receptors regulating transcription of several genes. In the MRL/*lpr* mouse model of lupus, vitamin A exerts paradoxical effects on the development of autoimmune lupus, resulting in decreasing inflammation in some organs or exacerbation of disease course in others (100). In another study with the (MRL/MPJ-*lpr/lpr*) SLE mouse model, vitamin A deficiency exerted a suppressive effect on the activities of abnormal T and B cells and suppressed serum anti-dsDNA antibody production (Figure 2) (101). Ikeda et al. (102) observed that 5–10 mg/kg oral administration of etretinate (a synthetic vitamin A derivative) significantly reduced dermal thickness (*p* < 0.05) in this SLE mouse model (MRL/*lpr*) compared with that of the controls by inducting apoptosis in dermal infiltrating cells and regulating cytokine production.

Considering human studies, Kinoshitak et al. (103) observed that after treatment with retinoids, levels of anti-dsDNA antibody and proteinuria were improved without side effects in patients with lupus nephritis. Daily intake of 100,000 U of vitamin A produced enhanced antibody-dependent cellular cytotoxicity, natural killer cell and IL-2 activities in patients with SLE (104). In a case-controlled study with Indonesian patients with SLE (*n* = 62), vitamin A modulated T-helper type (Th17), and Treg cell balance (105).

### Vitamin B

The role of the vitamin B group in autoimmune patients is usually discussed in the context of its insufficiency. Indeed, several studies have indicated a low level of vitamin B in SLE patients in comparison with those of healthy controls. Vitamin B_2_ (riboflavin) deficiency was detected in 88% of a cohort of Chinese patients with SLE (*n* = 258) in an observational study (106). Similarly, significantly low levels of vitamin B_12_ (cobalamin) were observed (<180 pg/mL) in another cohort of patients with SLE (*n* = 43) when compared with those of healthy individuals (*p* < 0.0005); however, this was not significantly associated with disease activity (107). Later, in a prospective study on female Japanese patients with SLE (*n* = 43) (98), an inverse correlation was observed between a high intake of vitamin B_6_ (pyridoxine) (1.7 mg/day) and the risk of active SLE, possibly through improving overall immune functions and suppressing active inflammation. Furthermore, immunotherapy with vitamin B_9_ (folate) alleviated SLE-related symptoms in two lupus-prone mouse models (NXBW/F1 and MRL/MpJ*Tnfrsf6*^*lpr*^), leading to a significant prolongation of survival (*p* = 0.005) in both cases (25–53 vs. 16–31 weeks for untreated mice) (108).

### Vitamin C

In a 4 year prospective study on Japanese patients with SLE (*n* = 279), it was observed that vitamin C intake (109.99 mg/day) was significantly inversely associated (*p* = 0.005) with the risk of developing active SLE (109). These researchers postulated that the antioxidant properties of vitamin C modulated immune functions, regulated the release of inflammatory mediators, decreased oxidative stress and suppressed autoantibody production in the SLE subjects. In a double-blind randomized placebo-controlled study of patients with SLE (*n* = 39), after a 12 week combined therapy with vitamin C (500 mg/day) and vitamin E (800 IU/day), reduction in lipid peroxidation was observed without affecting other oxidative stress markers or endothelial function (110).

### Vitamin D

Recently, the role of vitamin D in the development of various autoimmune diseases has attracted attention. Vitamin D is commonly recognized as a pleiotropic compound, and its role goes beyond managing calcium metabolism. Not long ago, it was confirmed that vitamin D exerted a strong effect on cellular proliferation, differentiation and immune modulation. Congruently, vitamin D exerted protective effects in several diseases like hypertension, diabetes, cardiovascular diseases (CVDs), autoimmune diseases and cancer development (111). Studies on the role of vitamin D are especially important because vitamin D deficiency is commonly observed in people from developed countries and may potentially contribute to the increasing burden of autoimmune diseases in the last few years (112, 113).

A significantly high prevalence of vitamin D deficiency has been observed in patients with SLE in Saudi Arabia (114), Bahrain (115), the USA (116, 117), Canada (118), Jamaica (119), Brazil (120), France (121), Hungary (122), Denmark (123), and Spain (124, 125). In clinical studies on patients with SLE, significant inverse correlations were observed between serum levels of vitamin D and SLE disease activity index (SLEDAI) score in Malaysian (126), Taiwanese (127), Thai (128), Indian (129), Egyptian (130, 131), Saudi Arabian (132), Brazilian (133, 134), Australian (135), American (136), Hungarian (122), and Chinese (137) populations with an exception of Serbian SLE subjects (*n* = 46) (138). Interestingly, two prolonged follow-up studies (up to 329 months) observed no associations between reported dietary intake of vitamin D during adolescence and risk of developing SLE in adulthood (139) and in adult women (140), suggesting that vitamin D metabolism may be at least partially related to inflammatory status in autoimmune patients. Several reasons for vitamin D inadequacy are independent of vitamin D dietary intake. Specifically, in lupus patients, vitamin D deficiency may be due to the avoidance of sunshine, photoprotection, impaired vitamin D synthesis (renal insufficiency) or the use of medications such as glucocorticoids, anticonvulsants, antimalarials, and calcineurin inhibitors, which exert a negative impact on the metabolism of vitamin D or downregulate the functions of the vitamin D receptor (113).

#### Vitamin D and SLE Activity

Considering the role of vitamin D deficiency on the development of SLE, Yu et al. (141) recently demonstrated that vitamin D plays a protective role in the autoantibody-induced injury of podocytes (kidney cells that wrap around capillaries of the glomerulus) in patients with lupus nephritis, which could be a potential novel therapeutic target to treat lupus nephritis. From a 24 week double-blind randomized placebo-controlled trial by Lima et al. (142), it was established that supplementation of vitamin D in patients with juvenile-onset SLE effectively improved parameters of bone microarchitecture (*p* = 0.024). Bone marrow mesenchymal stem cells from patients with SLE show impaired proliferative capacity compared with that of normal controls (143). Administration of an analog of vitamin D (EB1089) promoted proliferation and osteogenic differentiation of stem cells by the Smad 1/5/8 signaling pathway (144). Although vitamin D deficiency was insignificantly correlated with higher SLEDAI scores in Egyptian patients with juvenile-onset SLE (145), it was later suggested that vitamin D supplementation was effective for a significant reduction of SLEDAI (*p* = 0.01) and improving fatigue (*p* = 0.008) in Brazilian patients with juvenile-onset SLE (146). It was observed from two recent meta-analyses that the serum levels of vitamin D were significantly low (*p* < 0.00001) in patients with SLE compared with those of healthy controls (147), and vitamin D supplementation was significantly effective (*p* < 0.001) in increasing these low serum vitamin D levels in patients with SLE with improved fatigue (148). In a group of patients with SLE, levels of *mTOR* mRNA were higher in a group with severe vitamin D deficiency compared with a group with vitamin D insufficiency (*p* = 0.036) indicating that severe vitamin D deficiency contributes to SLE pathogenesis via increased expression of mTOR (149).

#### Cytokines and Innate Immunity

Increased levels of antinuclear antibodies, IL-23, and IL-17 (important for Th17 development and function) were also significantly associated (*p* < 0.05) with a vitamin D deficiency (120). Abnormal activation of toll-like receptors (TLRs) contributes to the pathogenesis of SLE, and, interestingly, vitamin D exerts some of its immunomodulatory effects in patients with SLE by significantly downregulating the expression levels of TLR3 (*p* = 0.03), TLR7 (*p* = 0.0001), and TLR9 (*p* = 0.007) (150). Autoimmune diseases like SLE may result from a conversion of Treg cells into Th17 cells, and in the serum of patients with SLE, IL-17 levels were abnormally high. After 6 months of vitamin D supplementation in a group of Portuguese patients with SLE (*n* = 24), the FoxP3^+^:IL-17A ratio was higher than that in the baseline (*p* < 0.001) (151). Similar conclusions arose from studies on IFNs. Low levels of vitamin D were significantly associated with high expression of IFN-α (*p* < 0.05) (130) and high levels of IFN-α (*p* = 0.02) (152) and IFN-γ (*p* = 0.03) (153) in the SLE group compared with the control group. Vitamin D deficiency was associated with impaired endothelial repair mechanisms and induction of CVDs with the potential to activate the type I IFN signaling pathway in SLE (154). Reynolds et al. (155) demonstrated that vitamin D positively modifies endothelial repair mechanisms by reducing *CXCL-10* expression and thus reduces risks of CVDs in SLE. Handono et al. (156) showed that vitamin D significantly prevented endothelial damage (*p* < 0.05) induced by increased neutrophil extracellular trap formation in patients with SLE.

#### Adaptive Immunity

Vitamin D also induced the expansion of SLE Treg cells (CD25^high^ Foxp3^+^). However, Schneider et al. (157) did not find any significant association between levels of vitamin D and cytokine profiles in SLE patients. After long-term (12 months) monthly treatment with vitamin D in patients with SLE, a significant enhancement (*p* = 0.03) of Treg cells (CD4^+^CD45RA^+^CCR7^−^) was observed (158). Similarly, lower levels of vitamin D caused a significant reduction (*p* = 0.015) in the percentage of migrated Treg cells (CD4^+^CD25^+^CCR4^+^) compared with that of healthy controls (159). The CD4^+^/CD8^+^ ratio was significantly higher (*p* < 0.001) in patients with severe vitamin D deficiency compared with a vitamin D-insufficient group (149), and vitamin D supplementation elevated CD4^+^CD8^+^ double-positive T cells (160) or inhibited the activation of CD4^+^ T cells (Figure 2) through inhibition of the PKCδ/ERK pathway and reduced expression of inflammatory factors (161). Through vitamin D supplementation for 6 months in patients with SLE (*n* = 20), hypovitaminosis D, Treg-cell balance (increased Treg cells and decreased effector Th1 and Th17 cells) and B-cell homeostasis were restored with reduced anti-dsDNA antibodies (162). Low levels of vitamin D (<20 ng/mL) were associated with reduced micro (mi)-RNA expression in T cells (*i.e*., miRNA-377, miRNA-342, miRNA-10a, miRNA-374b, miRNA-125a, and miRNA-410) of patients with SLE compared with those of healthy controls, indicating that unfavorable immunological alterations take place because of a lack of vitamin D in patients with SLE (163).

### Vitamin E

Considering the pivotal role of vitamin D in the development of SLE, data on the fat-soluble vitamin E are still scanty. Some animal studies have addressed this problem with contradictory results. In the one study using SLE-prone NZB/W F1 mice as a model, high-dose vitamin E supplementation (550 mg/kg all-rac-α-tocopheryl acetate) decreased anti-dsDNA IgG antibody (Figure 2), oxidative stress, regulated cytokines (IFN-γ and IL-6 secretion) and production of lymphocytes (B-cell differentiation), subsequently alleviating the severity of SLE with a prolonged lifespan (164). By contrast, Hsieh et al. (165) observed that low-dose vitamin E supplementation (250 mg/kg all-rac-α-tocopheryl acetate) was more beneficial for the survival of SLE-prone MRL/*lpr* mice, lowering the levels of anti-dsDNA, and anticardiolipin antibodies when compared with those of the high-dose group (500 mg/kg all-rac-α-tocopheryl acetate).

Our current understanding of the role of vitamin E in the development of SLE in humans is also limited. From a prospective case-control study, lower levels of serum vitamin E were detected in a group of SLE patients (0.64 ± 0.09 mg/dL) compared with those of normal controls (0.80 ± 0.21 mg/dL) (166). According to the study of Maeshima et al. (167), oral administration of vitamin E (150–300 mg/day) in patients with SLE suppressed synthesis of antibodies via a mechanism independent of antioxidant activity. In a study with Egyptian patients with SLE (*n* = 25) (168), when *Nigella sativa* and vitamin E were supplemented together for 3 months, levels of antioxidant enzymes such as reduced GSH and superoxide dismutase were increased significantly when compared with the levels before supplementation (*p* < 0.001). Moreover, levels of IL-10, malondialdehyde, nitric oxide and inducible nitric oxide synthase decreased significantly compared with pre-supplementation levels (*p* < 0.001). Additionally, there was a significant decrease in antinuclear antibodies (*p* < 0.001), anti-dsDNA levels (*p* < 0.001), and SLEDAI score (*p* < 0.01).

### Minerals

Like other micronutrients, minerals such as calcium, zinc, sodium, selenium, iron and copper generally attenuate SLE activity via different immunomodulatory mechanisms (Table 2). However, special attention must be given to mineral intake because it is best to restrict the consumption of some minerals like sodium. Table 2 shows the major dietary mineral sources based on the *United States Department of Agriculture (USDA), National Nutrient Database for Standard Reference* (60).

**Table 2:** Effects of dietary minerals on disease activities of SLE.

**Minerals**	**Study model**	**Country**	**Findings**	**Year, References**
Calcium	Cross-sectional	Spain	Vitamin D-calcium supplementation increased the serum levels of vitamin D, however, do not modify the serum calcium levels rather increased arterial stiffness significantly (IMT; *p* = 0.041).	2019, (169)
	Prospective interventional	Saudi Arabia	Vitamin D-calcium supplementation significantly improved the bone mineral density in vitamin D-deficient SLE patients, however, not significantly attenuated immune markers or disease activity.	2018, (170)
	Case-control	Egypt	There was no significant correlation between SLEDAI score and calcium supplementation (*p* = 0.861).	2016, (171)
	Animal model	USA	In presence of high-calcium diet, vitamin D supplement markedly suppress inflammatory T-cell activity in experimental MRL/*lpr* SLE mice.	2001, (172)
Zinc	Animal model	USA	Zinc restriction reduced autoantibodies (*i.e*., anti-dsDNA antibody) and lymphoproliferation in MRL/*lpr* SLE mice.	2001, (173)
	Animal model	USA	Zinc-deficient diet retarded autoantibody (*i.e*., anti-dsDNA antibody) production in NZB/NZW SLE mice.	1982, (174)
	Animal model	USA	Depot-zinc therapy significantly reduced kidney damage in the B/W SLE mice (*p* < 0.01).	1981, (175)
Sodium	Cross-sectional	Mexico	A positive correlation was detected between sodium intake and levels of CD4^+^CD25^+^Foxp3^+^ Treg cells in SLE.	2018, (176)
	Prospective	Italy	Due to dietary sodium intake, Th17 and Treg cells significantly decreased (*p* = 0.01) and increased (*p* = 0.04), respectively. Additionally, serum IL-9 levels were significantly reduced in SLE patients (*p* = 0.03).	2017, (177)
	Animal model	China	Excessive intake of sodium in diet aggravated lupus nephritis through SGK1 pathway by significantly increasing the Th1/Th2 and Th17/Treg ratios in MRL/*lpr* SLE mice.	2015, (178)
Selenium	Animal model	USA	Selenium supplementation leads to impaired differentiation and maturation of macrophages.	(179)
			Selenium in the drinking water significantly improved the survival rate (*p* < 0.04) and increased NK cell activity (*p* < 0.001) of the NZB/NZW SLE mice though there was no effect on autoantibody (*i.e*., anti-ssDNA antibody) production.	1988, (180)
Iron	Animal model	USA	Anemia and incidence of skin lesions were high in severely iron deficient MRL/MPJ-*lpr*/*lpr* SLE mice.	1995, (181)
Copper	Double blind, double placebo-controlled trial	Ireland	No significant effect on SLAM-R was observed.	2004, (182)

*SLEDAI, Systemic lupus erythematosus disease activity index; dsDNA, Double stranded DNA; ssDNA, Single stranded DNA; IMT, Intima-media thickness; SGK1, Serine/threonine protein kinase 1; Th, T helper; Treg, Regulatory T; IL, Interleukin; NK, Natural killer; SLAM-R, Revised Systemic Lupus Activity Measure*.

## Nutraceutics

Evidence accumulated over the past decade has demonstrated that various nutrients possess additional effects on body function and metabolism. Concordantly, several studies have shown that nutrients modify the immune response as well as the integrity of the organs and tissues. In connective tissue diseases where a pharmacological approach is limited and introduces the risk of several severe side effects, there is a demand for compounds that potentially exert beneficial effects without side effects. Such compounds are commonly referred to as nutraceuticals, and this approach, although a bit controversial, has been tested in several SLE studies.

### Polyphenols

#### Curcumin

Curcumin, a major natural polyphenol of turmeric, exerted a protective effect against lupus nephritis in SLE-prone MRL/*lpr* mice by reducing serum anti-dsDNA antibody levels and inhibiting the expression of the NLRP3 inflammasome, which is believed to be a key player in the development of lupus nephritis (183). An experimental study with SLE-prone MRL/*lpr* mice demonstrated that curcumin aggravated CNS pathology (184). Moreover, curcumin decreased anti-dsDNA IgG antibodies and attenuated lupus nephritis upon interaction with Treg cells in SLE-prone NZB/W F1 female mice (185).

Direct translation of these promising data into clinical application is a difficult task; data in this field are scarce and frequently controversial. In a small double-blind randomized controlled trial (*n* = 39), when curcumin was administered along with vitamin D in a group of patients with SLE who displayed hypovitamin D, no significant differences arose in SLEDAI reduction or decreased serum IL-6 and increased TGF-β1 (186). Contrary to this, another study revealed that administration of low doses of curcumin (0.1 and 1.0 μg/mL) to cultured CD4^+^ cells from patients with SLE significantly (*p* < 0.001) modulated the Th17/Treg balance without affecting healthy subjects (187). Similar to this *in vitro* experiment, low-dose curcumin (0.1 μg/mL) inhibited the expression and activation of *PYK2* in peripheral blood mononuclear cells (PBMCs) and reduced the proliferation of PBMCs in patients with lupus nephritis (188).

Although the results of completed studies are somewhat promising, they are inconclusive, and more studies on the role of curcumin in SLE are warranted.

#### Virgin Olive Oil

In a pristane-induced BALB/c mouse model of SLE, administration of virgin olive oil (VOO) and its phenol fraction (with major components of hydroxytyrosol, tyrosol, oleuropein aglycone, and ligstroside aglycone) counteracted inflammatory pathways in cells of the monocyte–macrophage lineage (189). Therefore, both VOO and its phenol fraction are promising immunomodulators of SLE activity. Epigallocatechin-3-gallate (EGCG), the major bioactive polyphenol present in green tea, enhanced the Nrf2 antioxidant signaling pathway, decreased renal NLRP3 inflammasome activation and increased systemic Treg cell activity in NZB/W F1 SLE-prone mice (190). EGCG attenuated inflammation in mesangial cells of SLE-prone MRL/*lpr* mice via the PI3K/Akt/mTOR pathway by decreasing Akt phosphorylation (191).

Limited data exist on the role of VOO in modulating the immune response in SLE patients. An *in vitro* study on PBMCs derived from SLE patients (192) showed that the phenol fraction of VOO was successful in modulating cytokine production and attenuated induced T-cell activation, most likely through the NF-κB signaling pathway.

#### Polyphenols and Microbiota

Cuervo et al. (193) determined an association between polyphenol intake and fecal microbiota in Spanish patients with SLE compared with healthy controls. Major dietary sources of polyphenols include various fruits and vegetables (oranges, lettuce, watermelon, kiwi, tomato, apple, lentils and celery) as assessed through a semi-quantitative food-frequency questionnaire. Interestingly, a well-balanced diet with a high intake of apples and oranges besides other fruits and vegetables rich in flavonoids has been associated with the presence of beneficial microorganisms (*i.e*., *Lactobacillus, Blautia*, and *Bifidobacterium*) in the fecal matter of these patients with SLE.

#### Flavonoids

The results of a 6 year survey collected in 2016 showed that higher serum levels of lycopene (enriched in tomatoes, red carrots and watermelons) have a significant protective effect on mortality when compared with lower levels (5.3 vs. 33.3%; *p* = 0.04) in subjects with SLE (194). Apigenin (a mutagenic flavonoid rich in parsley, thyme, peppermint, olives and chamomile) inhibited autoantigen presentation and stimulatory functions of antigen-presenting cells necessary for activation and expansion of autoreactive Th1 and Th17 cells (Figure 2) and B cells in SLE by downregulation of COX-2 expression (195). Nevertheless, Pocovi-Gerardino et al. (196) documented no significant correlation between the dietary intake of macronutrients, micronutrients or antioxidants, and serum levels of CRP in patients with SLE.

### Royal Jelly

Royal jelly, a creamy product secreted by young nurse worker bees (*Apis mellifera*), is composed of 10–12% carbohydrates, 12–15% proteins, and 3–7% lipids, vitamins, minerals and polyphenols. In an open-label study, children with SLE (*n* = 20) received 2 g of freshly prepared royal jelly daily for 12 weeks. The SLEDAI score significantly improved (*p* = 0.01) after 3 months of royal jelly therapy. Complement C3 (*p* = 0.009) and C4 (*p* = 0.014) levels also significantly increased at the end of the 12-week treatment. Additionally, percentages of CD4^+^CD25^+high^ and CD4^+^CD25^+high^FOXP3^+^ cells (CD4^+^ Treg cells) were significantly increased after royal jelly treatment (*p* < 0.01); however, the frequency of Treg cells remained significantly lower than that in the control group (*p* = 0.01). Moreover, CD8^+^CD25^+high^ and CD8^+^CD25^+high^FOXP3^+^ cells (CD8^+^ Treg cells) significantly increased after royal jelly treatment (*p* < 0.01). Percentages of CD8^+^CD25^+high^ and CD8^+^ Treg cells in patients with SLE at baseline (prior to royal jelly treatment) were significantly lower than in the control group of children without SLE (*p* = 0.003) (197).

In another study, when royal jelly was administered in a group of SLE-prone NZB/NZW F1 female mice, a significant delay in the onset of the disease and prolonged lifespan were observed. Additionally, serum levels of anti-ssDNA, anti-dsDNA antibodies and autoreactive B cells were significantly decreased (198).

## Conclusions

Because dietary supplementation of various macronutrients and micronutrients has exhibited immunomodulatory effects including maintenance of homeostasis and improvement of physical and mental well-being of patients with SLE, it is recommended that these patients consume a balanced diet that is low in calories and protein but contains plenty of fiber, PUFAs (ω-3 and ω-6), vitamins (A, B, C, D, and E), minerals (calcium, zinc, selenium, iron and copper) and polyphenol-containing foods.

## Author Contributions

MI contributed to the conception of the work, developed the search strategy, conducted the literature search and drafted the manuscript. SK assisted in writing and drawing Figure 2. PK and RH substantively revised the manuscript. All the authors approved the final submitted version of the manuscript.

## Conflict of Interest

The authors declare that the research was conducted in the absence of any commercial or financial relationships that could be construed as a potential conflict of interest.
