# Supplementary material for: Immunomodulatory Effects of Diet and Nutrients in Systemic Lupus Erythematosus (SLE): A Systematic Review
Source: Front Immunol. 2020 Jul 22;11:1477. doi: 10.3389/fimmu.2020.01477 (PMC7387408; doi:10.3389/fimmu.2020.01477)
Supplement: Supplementary file 1 [file Data_Sheet_1.DOCX]

**Appendix**

**Search Strategy**

**PubMed**

((((SLE[Title] OR "Systemic lupus erythematosus"[Title] OR lupus[Title])) AND (food[Title] OR nutrient*[Title] OR diet[Title] OR intake[Title] OR antioxidant*[Title] OR benefit*[Title] OR nutrition*[Title] OR physicochemical[Title] OR dietary[Title] OR bioactive[Title] OR composition[Title] OR supplement*[Title] OR vitamin*[Title] OR mineral*[Title] OR phenol*[Title] OR “olive oil”[Title] OR curcumin[Title])))

**Google Scholar**

allintitle:(SLE OR "Systemic lupus erythematosus" OR lupus) (food OR foods OR nutrient OR nutrients OR diet OR intake OR antioxidant OR antioxidants OR nutrition OR benefit OR nutritional OR physicochemical OR dietary OR bioactive OR composition OR supplement OR supplements OR vitamin OR vitamins OR mineral OR minerals OR phenol OR phenols OR “olive oil” OR curcumin)

**Scopus**

TITLE(SLE OR "Systemic lupus erythematosus" OR lupus) AND TITLE(food OR foods OR nutrient OR nutrients OR diet OR intake OR antioxidant OR antioxidants OR nutrition OR benefit OR nutritional OR physicochemical OR dietary OR bioactive OR composition OR supplement OR supplements OR vitamin OR vitamins OR mineral OR minerals OR phenol OR phenols OR “olive oil” OR curcumin)
